# Supplementary material for: Short-term periodic restricted feeding elicits metabolome-microbiome signatures with sex dimorphic persistence in primate intervention
Source: Nat Commun. 2024 Feb 5;15:1088. doi: 10.1038/s41467-024-45359-z (PMC10844192; doi:10.1038/s41467-024-45359-z)
Supplement: Supplementary file 1 — Supplementary Information [file 41467_2024_45359_MOESM1_ESM.pdf]

Supplementary Figure 1

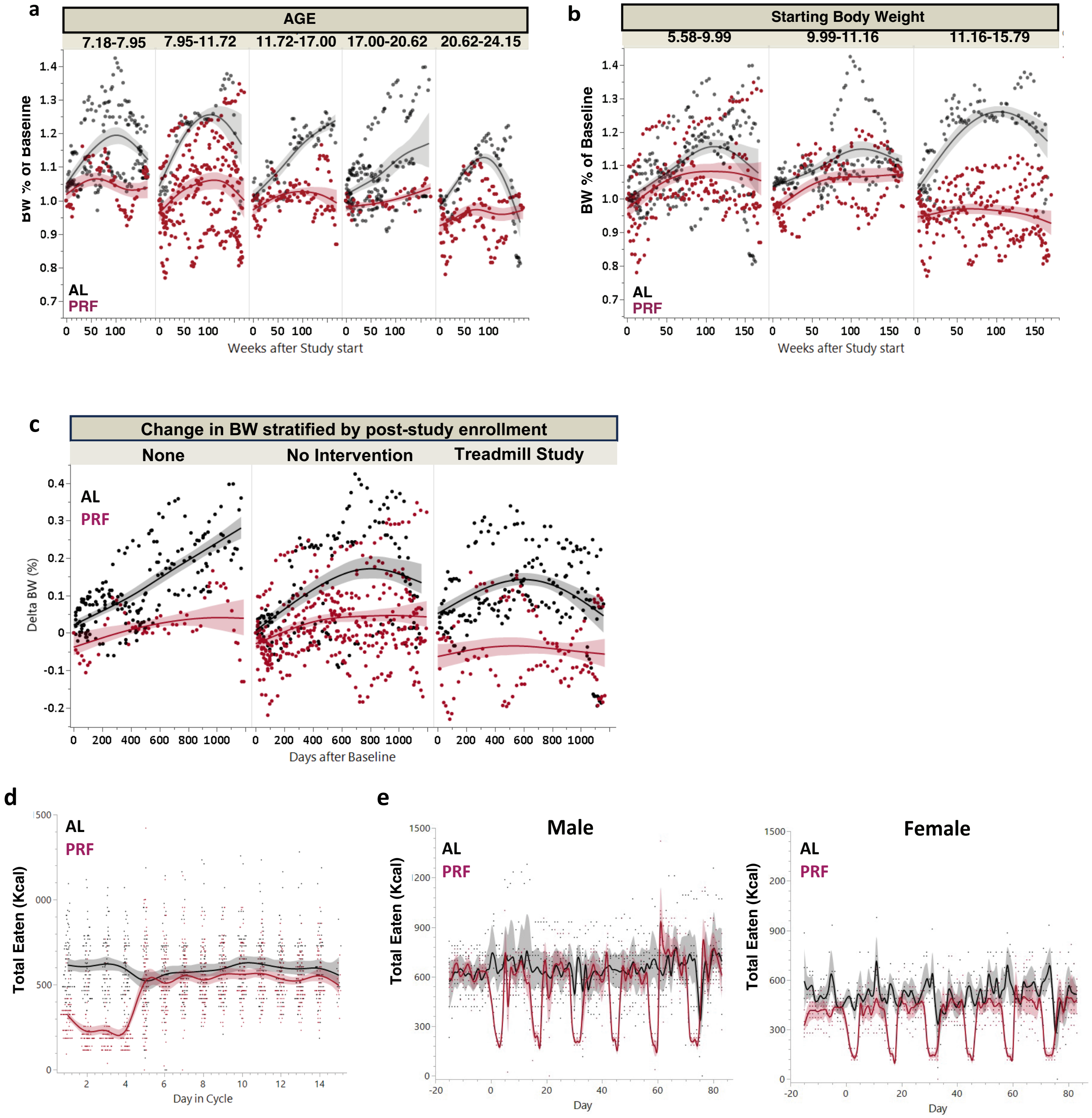

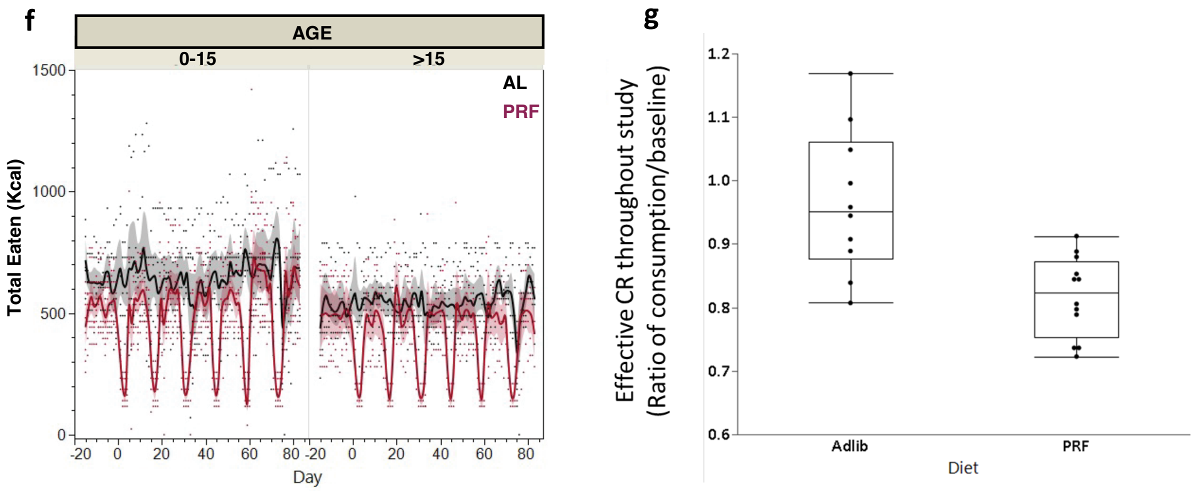

Supplementary Figure 1. Body weight and food intake by various groupings. a Long-term body weight follow-up stratified by age groups. Age groups are indicated in the top (years). Each dot represents a single animal. Line denotes a spline regression (λ = 0.5). **b** Long term body weight follow-up stratified by starting body weight. **c** Long term body weight follow-up stratified by animal assignment to other studies after conclusion of diet. No additional study enrollment, non-intervention enrollment, treadmill study enrollment. **d** Food uptake follow-up as a function of day in the diet cycle. Shown are all cycles concatenated together where each dot represents a single animal daily measurement. The line represents the spline regression (λ = 0.5) with confidence of fit (shadow). **e** Daily food consumption stratified by sex. **f** daily food consumption stratified by age (indicated on top in years). **g** Effective CR measured as average daily food consumption throughout the study divided by the daily average baseline amounts for each individual animal. Quartile boxes are presented with mean value.

Supplementary Figure 2

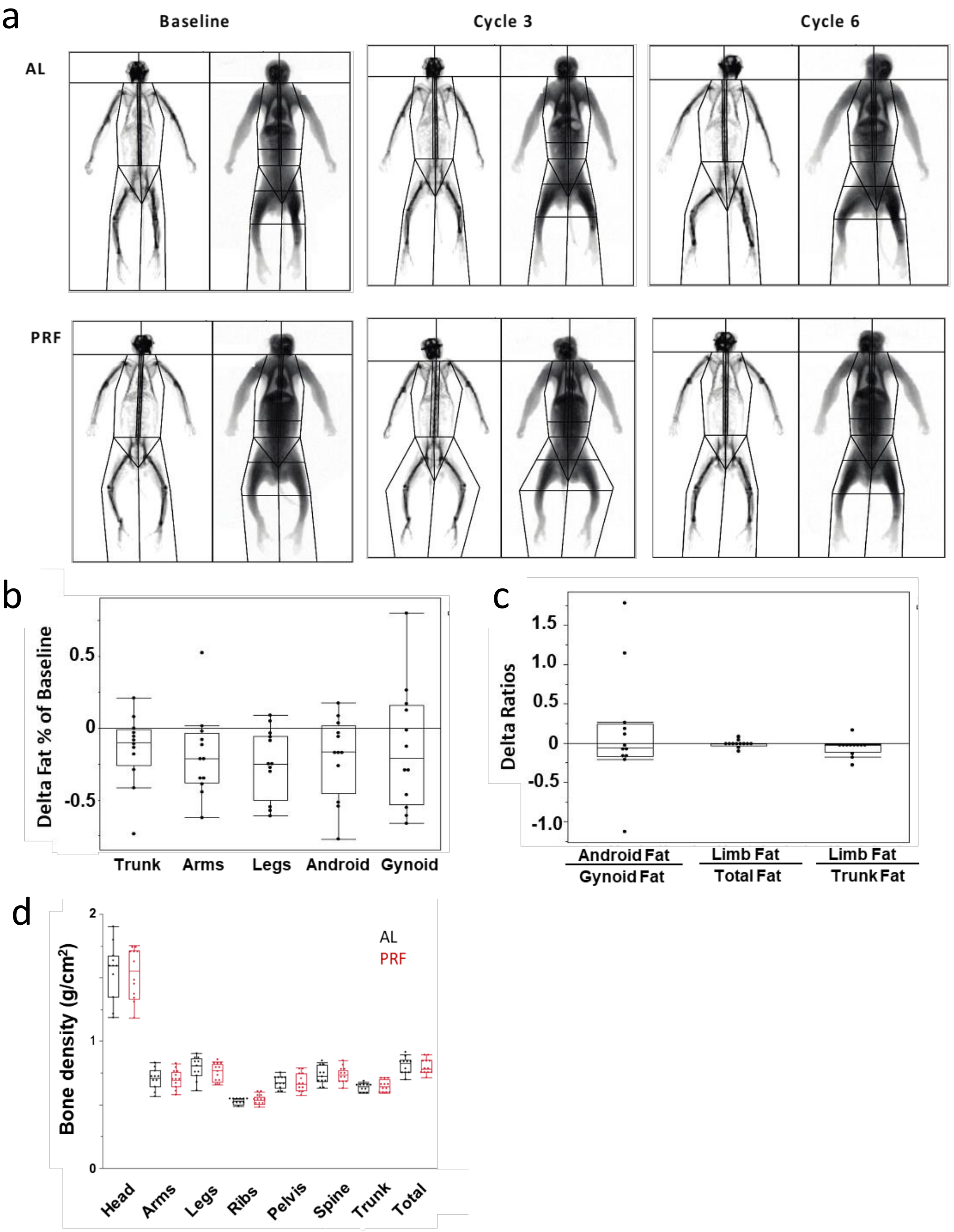

Supplementary Figure 2. DEXA analysis. a Representative DEXA scans using the GE Lunar Prodigy DXA. **b, c** Changes in fat% between study start and end in animals under PRF diet. **d** Bone density measure for the depicted body parts at the end of the study. Each dot represents a single animal, and the box denotes the quartiles with median bar. Whiskers are expected variation as calculated by 1.5 times the interquartile range

Supplementary Figure 3

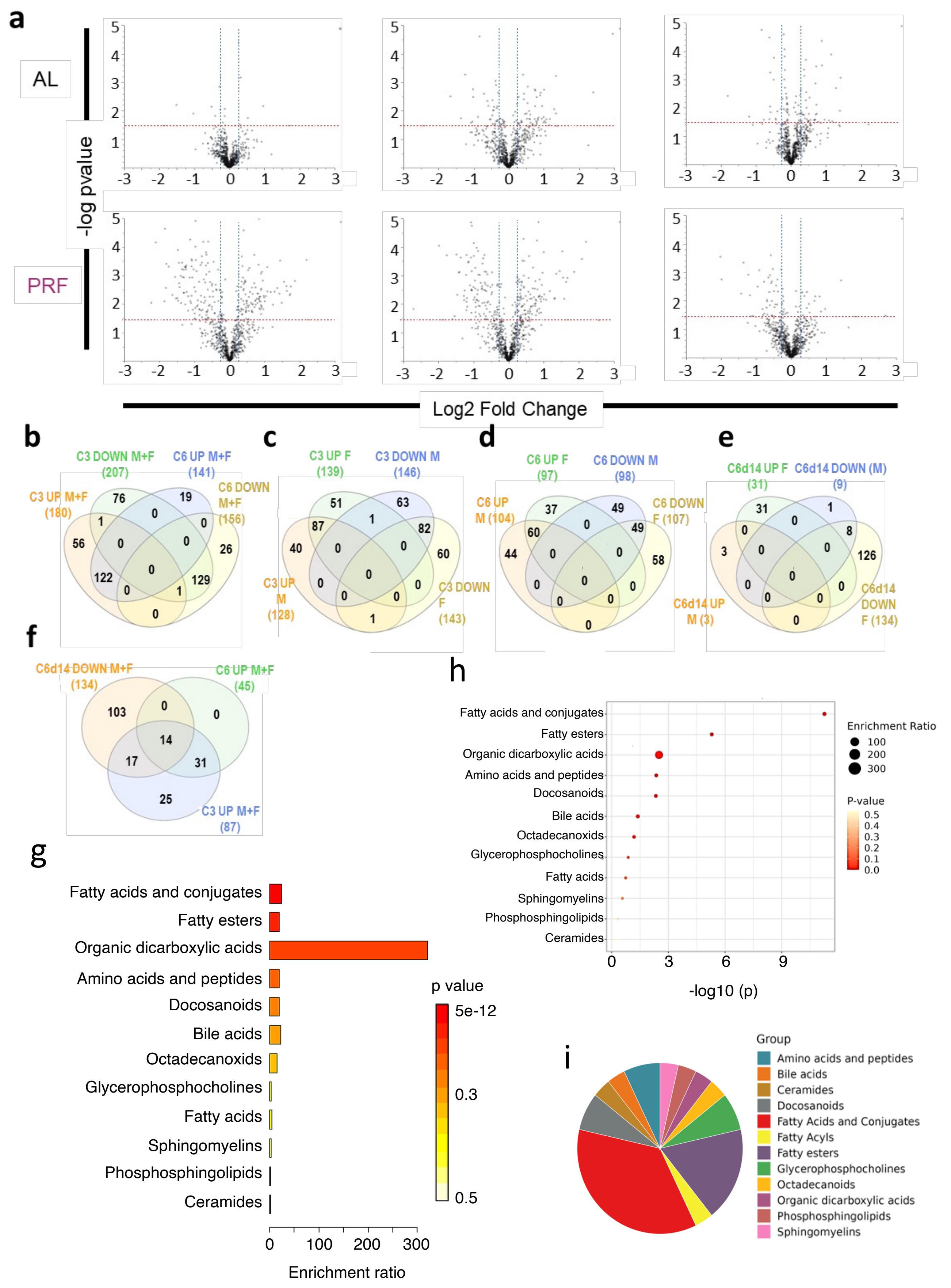

Supplementary Figure 3. Metabolomics. a Volcano plots of metabolome changes during the study at the indicated timepoints for AL and PRF animals. Statistical significance is denoted with a red line and impactful fold change by blue lines. **b,c,d,e, f** Venn diagrams showing commonalties in differentially abundant serum metabolites ($p<0.05$, $q<0.1$, $FC>|1.3|$) for the indicated comparisons. **g, h, i** analysis for all upregulated serum metabolites during peak diet (both cycles aggregated) with an enrichment analysis (**g**), enriched groups (**h**), and distribution by metabolite type (**i**).

Supplementary Figure 4

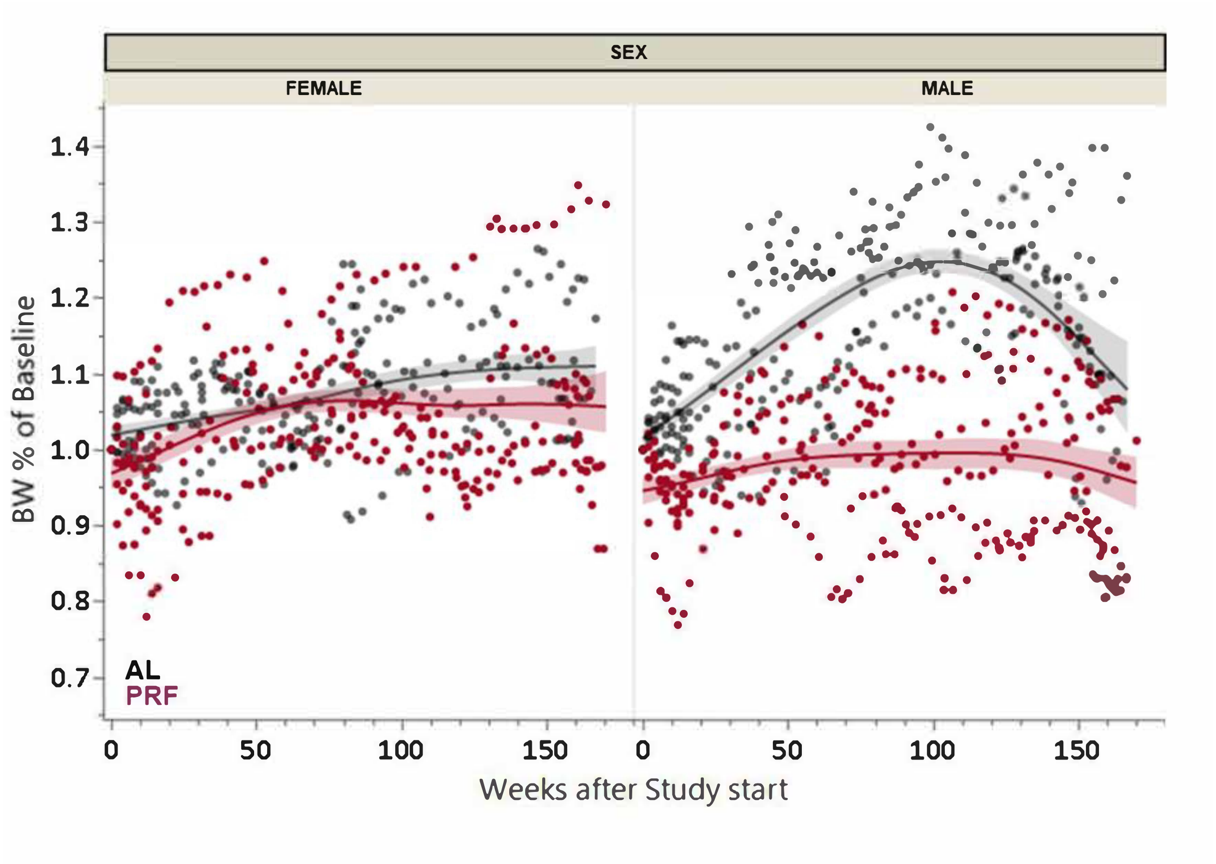

Supplementary Figure 4. Stratification of long-term body weight follow-up by sex. Body weight presented as percent of baseline body weight stratified by sex. Each dot represents a single measurement, the group line was calculated with a spline function (λ = 0.5) and the shaded area represents the confidence of fit with color corresponding to experimental group.

Supplementary Figure 5

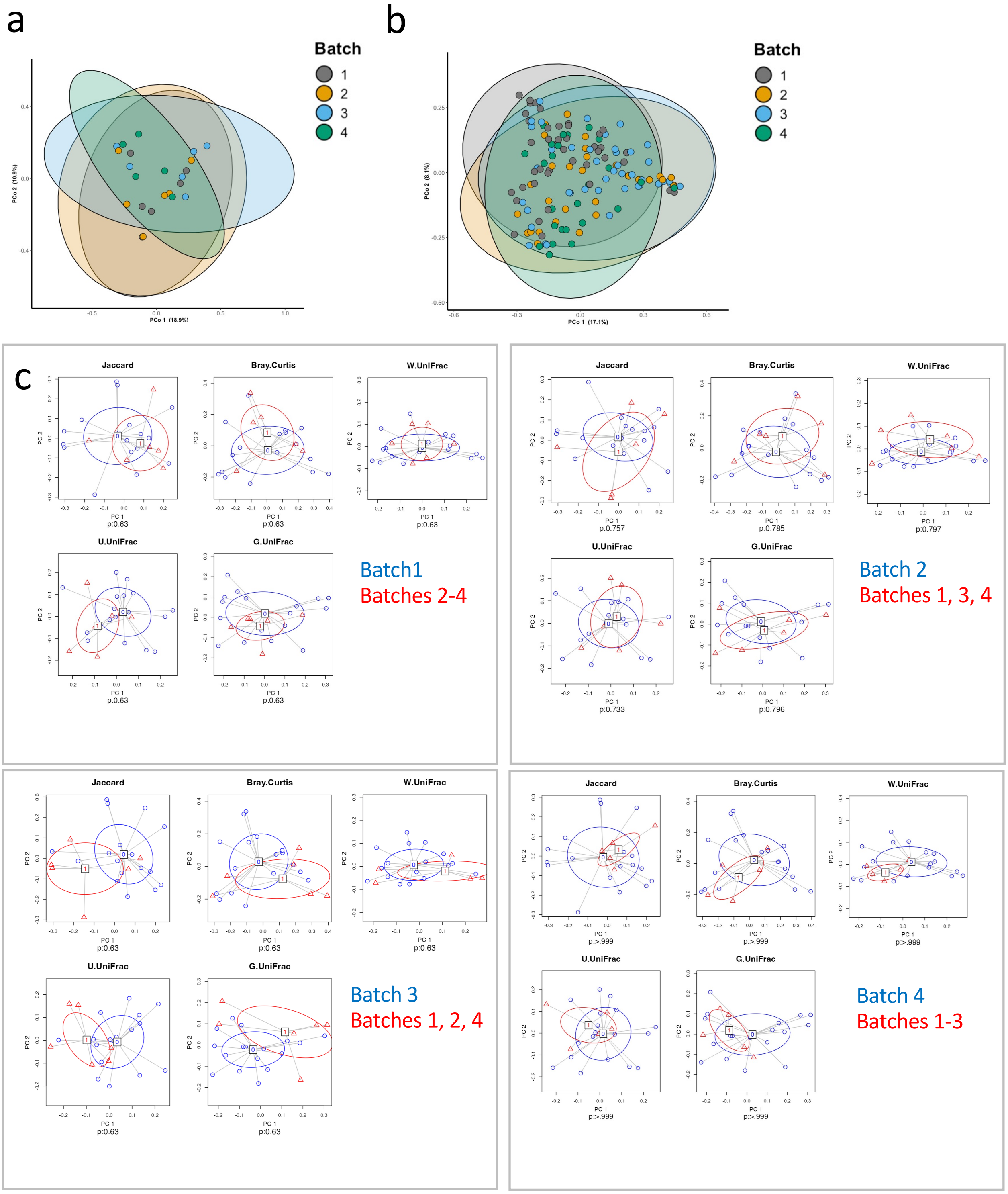

Supplementary Figure 5. Batch effect test in the gut microbiome. a PCoA plots of all animals at baseline colored by batch as in b. **b** PCoA of all samples colored by batch. **c** beta-diversity test between each batch and all other batches.

Supplementary Figure 6

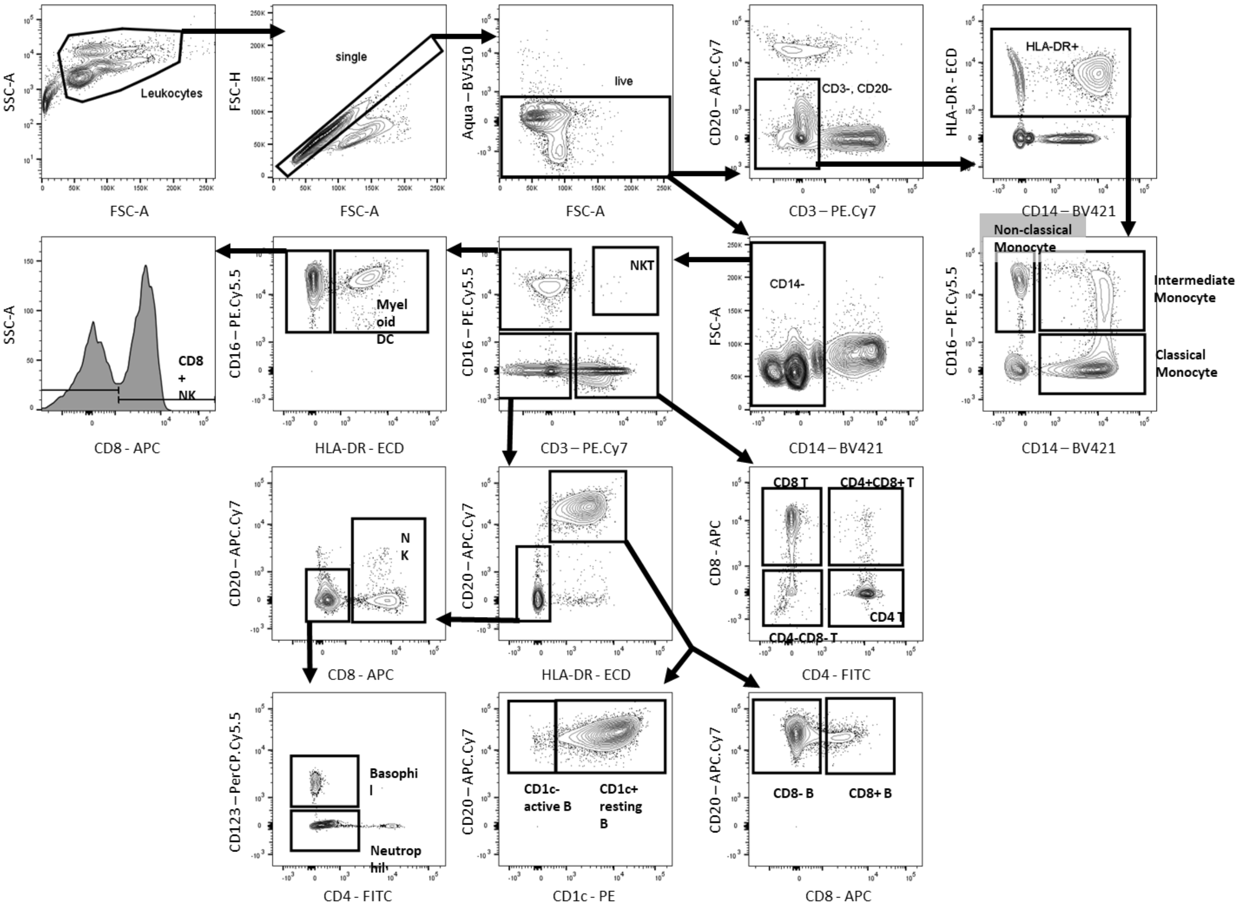

Supplementary Figure 6. Gating strategy of flow cytometry. Shown is a representative sample for blood cell analysis

Supplementary Figure 7

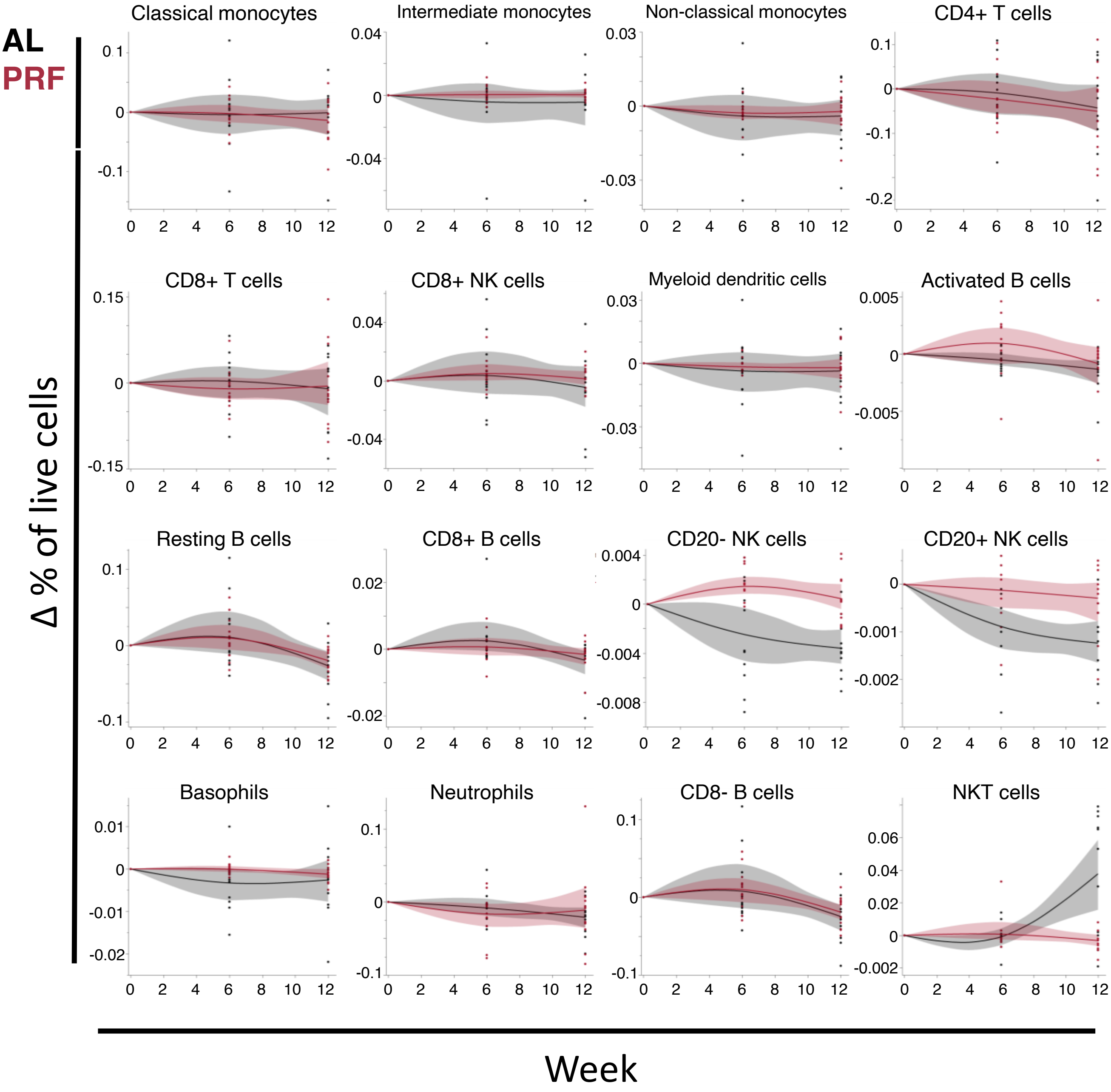

Supplementary Figure 7. Change from baseline of immune cell population frequencies quantified by flow cytometry. Blood parameters presented as change from baseline at measured timepoints, 6 weeks and 12 weeks. Each dot represents a single subject and lines represent a spline regression ($\lambda = 0.5$) and the shaded area represents the confidence of fit with color corresponding to experimental group.

Supplementary Figure 8

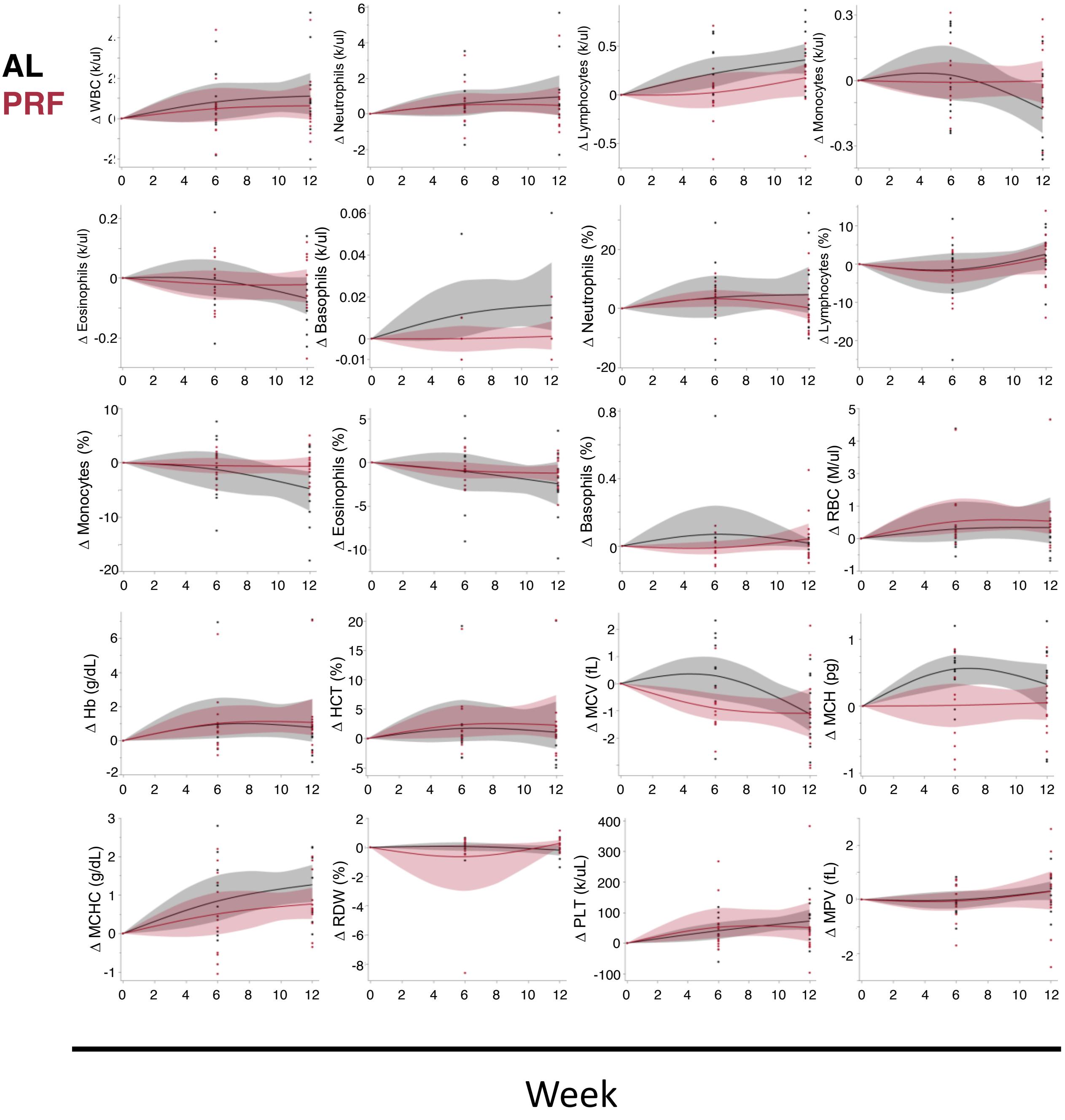

Supplementary Figure 8. Change from baseline of complete blood cell count data. Blood parameters presented as change from baseline at measured timepoints, 6 weeks and 12 weeks. Each dot represents a single subject and lines represent a spline regression (λ = 0.5) and the shaded area represents the confidence of fit with color corresponding to experimental group

Supplementary Figure 9

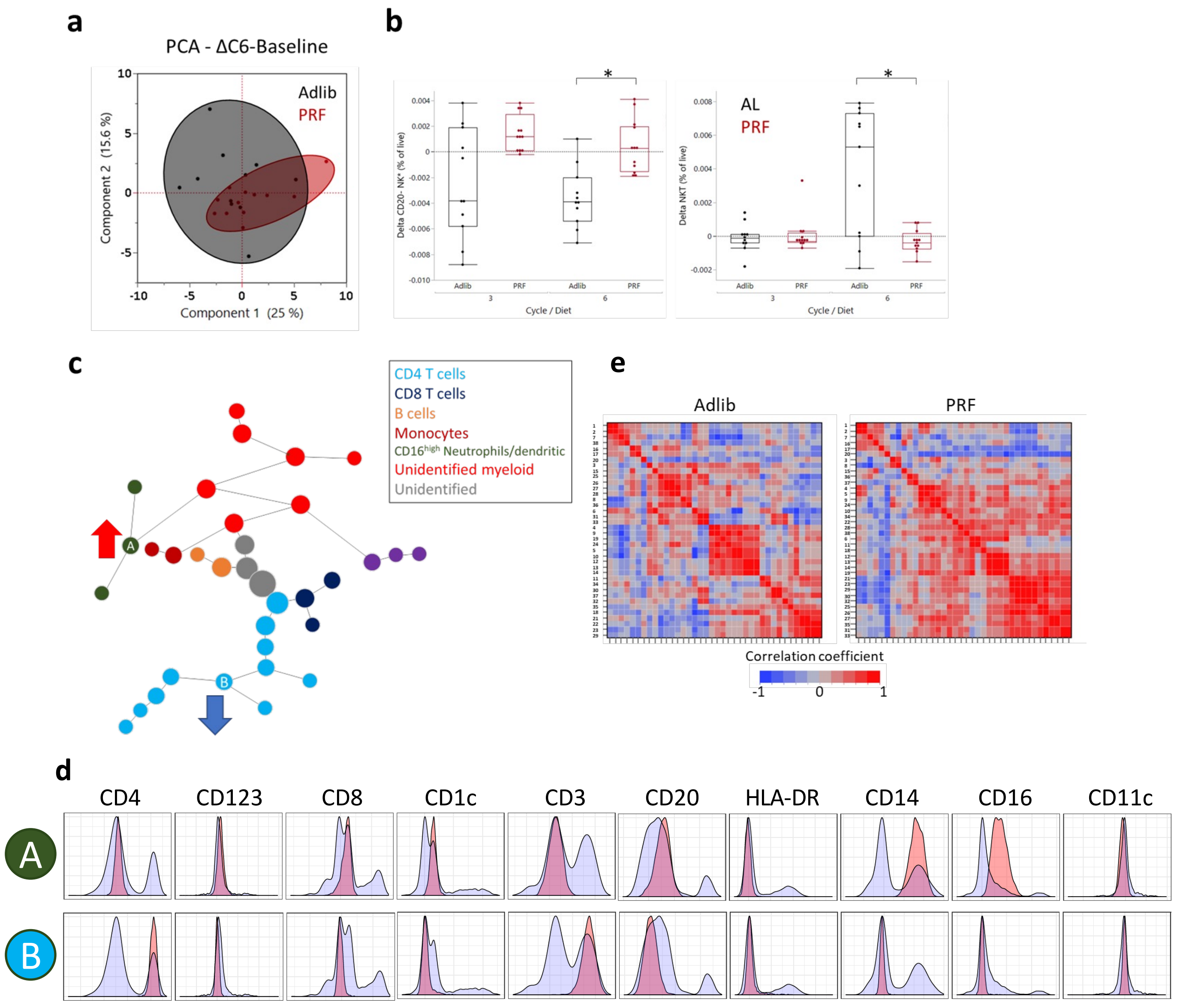

Supplementary Figure 9. Peripheral blood analysis. a Principal component analysis (PCA) of all acquired blood parameters (n=36). **b** Frequencies of the indicated NK cell populations as delta from baseline. Each dot represents a single animal, and the box denotes the quartiles with median bar. Whiskers are expected variation as calculated by 1.5 times the interquartile range. Statical analysis was measured by unpaired t-test * p<0.05 **c** Results of Citrus analysis. All flow cytometric data was analyzed on the Cytobank platform with the Citrus tool on default settings. Presented is the summary of the analysis in the form of a population tree with indicated changes to subpopulations of cd16^{high} neutrophils ("A") and CD4 T-cells ("B"). **d** Histogram of expression of the indicated antibodies for the Citrus analysis for the indicated clusters. **e** . Correlation matrix of changes observed for all blood parameters measured for the AL (left) and PRF (right) animals.

Supplementary Figure 10

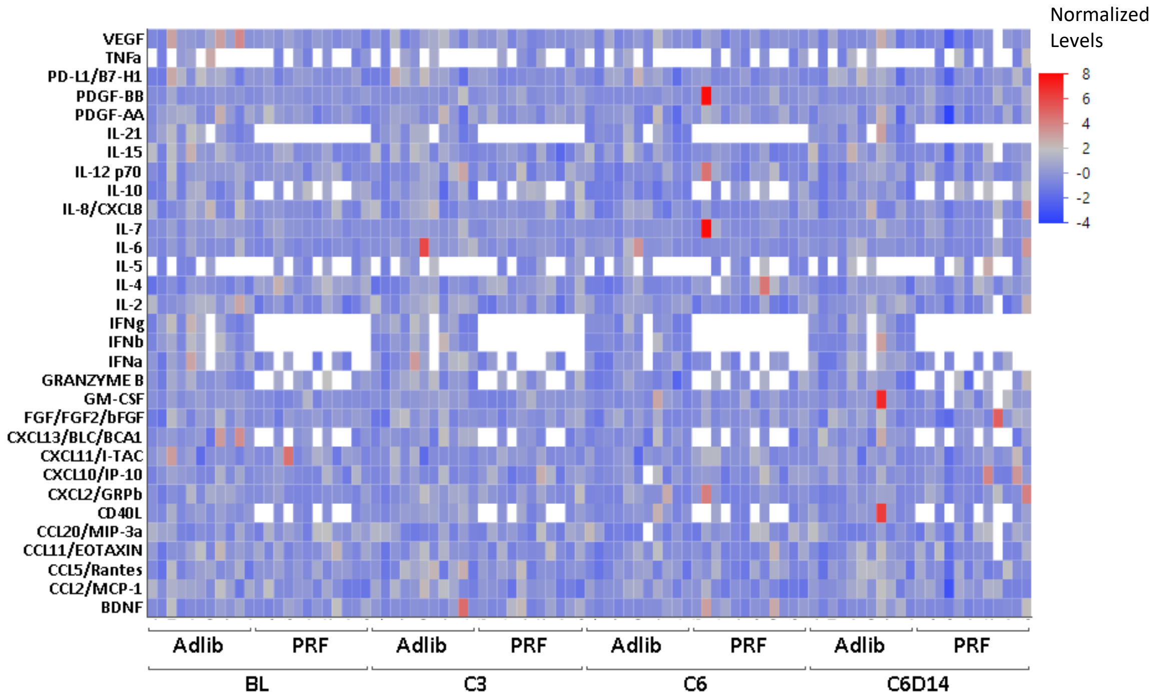

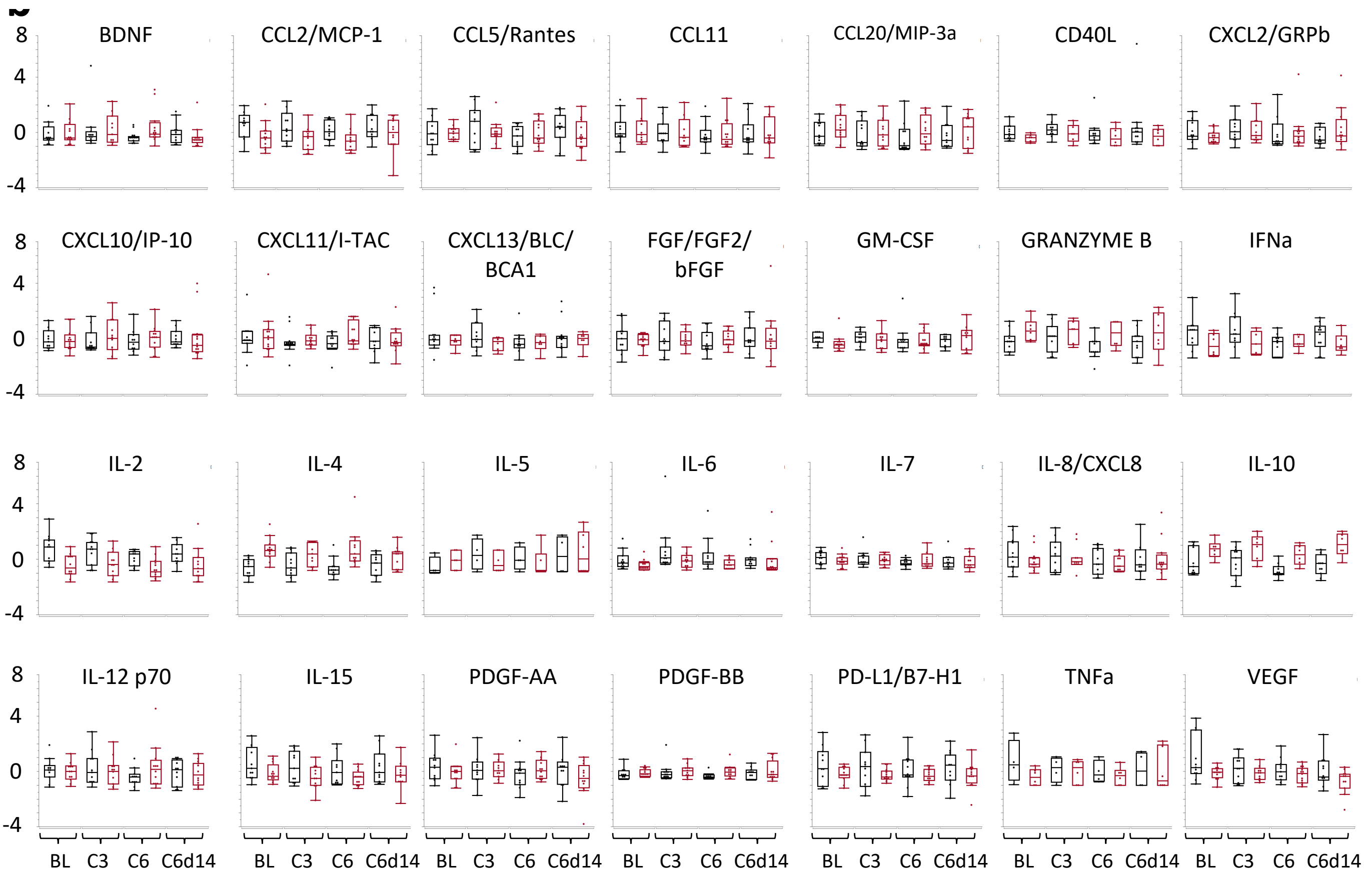

Supplementary Figure 10. Blood serum cytokine levels. Blood serum was collected and stored frozen until cytokine levels were quantified using the Luminex Performance cytokine panel adjusted for NHPs. **a** heatmap of normalized data for visualization purposes, each column represents a single animal under the indicated diet and timepoint (BL=baseline, C3=cycle 3 day 4, C6=cycle 6 day 4, C6d14=cycle 6 day14. Each row shows a single cytokine. White spaces indicate missing values. **b** Individual cytokine levels presented as a dot plot with quartile box plots. Each dot represents a single animal with median bar. Whiskers are expected variation as calculated by 1.5 times the interquartile range. Omitted are the cytokines with a high number of missing values (IFNb, IFNg, and IL-21). AL=black, PRF=red.

Supplementary Figure 11

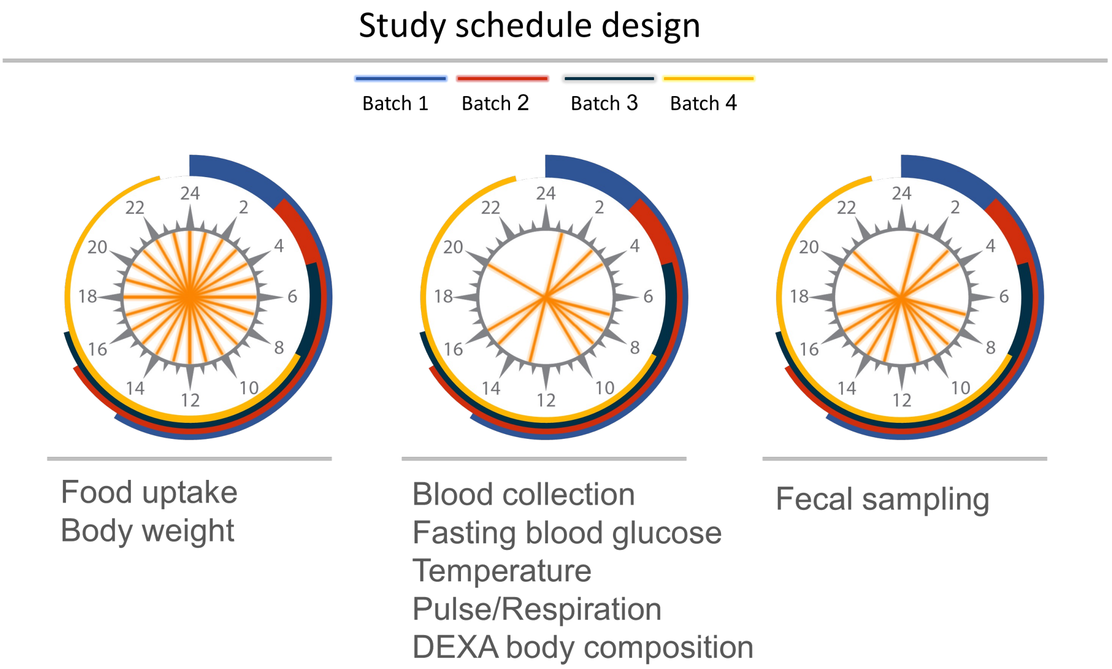

Supplementary Figure 11. Study design schedule schematic. Presented are graphical clocks denoting weeks after study start. Each batch is presented by a different color for the duration of measurement. Each measurement is listed below the time of collection
