# Supplementary material for: Short-term periodic restricted feeding elicits metabolome-microbiome signatures with sex dimorphic persistence in primate intervention
Source: Nat Commun. 2024 Feb 5;15:1088. doi: 10.1038/s41467-024-45359-z (PMC10844192; doi:10.1038/s41467-024-45359-z)
Supplement: Supplementary file 3 — Description of Supplementary Data [file 41467_2024_45359_MOESM3_ESM.docx]

**Supplementary Data 1. Differential abundance analysis for serum metabolites.**

Presented are all measured metabolites and their differential abundance test scores in terms of fold change (FC), p-value (p-val), and False Discovery Rate corrected p-value (q-val). Each row represents a single metbolite. Presented is the data for males (M), females (F), baseline, cycle 3 day 4 (C3), cycle 6 day 4 (C6), and cycle 6 day 14 (C6d14). Statistics were calculated using a 2 sided t-test.

**Supplementary Data 2. List of pathways enriched in the gut microbiome during PRF diet.** Shown are all enrichment scores for pathways that are enriched during peak diet in the gut microbiome. Statistical evaluation of enrichment was performed using Fisher's exact test.
