# Supplementary material for: Short-term periodic restricted feeding elicits metabolome-microbiome signatures with sex dimorphic persistence in primate intervention
Source: Nat Commun. 2024 Feb 5;15:1088. doi: 10.1038/s41467-024-45359-z (PMC10844192; doi:10.1038/s41467-024-45359-z)
Supplement: Supplementary file 6 — Reporting Summary [file 41467_2024_45359_MOESM6_ESM.pdf]

nature portfolio

Corresponding author(s): Isabel Beerman

Last updated by author(s): Dec 17, 2023

# Reporting Summary

Nature Portfolio wishes to improve the reproducibility of the work that we publish. This form provides structure for consistency and transparency in reporting. For further information on Nature Portfolio policies, see our Editorial Policies and the Editorial Policy Checklist.

## Statistics

For all statistical analyses, confirm that the following items are present in the figure legend, table legend, main text, or Methods section.

| n/a | Confirmed | |
|---|---|---|
| ☐ | ☒ | The exact sample size (*n*) for each experimental group/condition, given as a discrete number and unit of measurement |
| ☐ | ☒ | A statement on whether measurements were taken from distinct samples or whether the same sample was measured repeatedly |
| ☐ | ☒ | The statistical test(s) used AND whether they are one- or two-sided<br>*Only common tests should be described solely by name; describe more complex techniques in the Methods section.* |
| ☐ | ☒ | A description of all covariates tested |
| ☐ | ☒ | A description of any assumptions or corrections, such as tests of normality and adjustment for multiple comparisons |
| ☐ | ☒ | A full description of the statistical parameters including central tendency (e.g. means) or other basic estimates (e.g. regression coefficient) AND variation (e.g. standard deviation) or associated estimates of uncertainty (e.g. confidence intervals) |
| ☐ | ☒ | For null hypothesis testing, the test statistic (e.g. $F$, $t$, $r$) with confidence intervals, effect sizes, degrees of freedom and $P$ value noted<br>*Give P values as exact values whenever suitable.* |
| ☒ | ☐ | For Bayesian analysis, information on the choice of priors and Markov chain Monte Carlo settings |
| ☐ | ☒ | For hierarchical and complex designs, identification of the appropriate level for tests and full reporting of outcomes |
| ☒ | ☐ | Estimates of effect sizes (e.g. Cohen's $d$, Pearson's $r$), indicating how they were calculated |

*Our web collection on statistics for biologists contains articles on many of the points above.*

## Software and code

Policy information about availability of computer code

| | |
|---|---|
| Data collection | No software was used |
| Data analysis | FlowJo (BD, version 10), JMP (JMP Statistical Discovery LLC, version 17), R (2023) with the following packages: QIIME2, DADA2, USEARCH, aMiAD, Fossil, Picante, Entropart, PICRUSt2. Python (version 3.12.1). |

For manuscripts utilizing custom algorithms or software that are central to the research but not yet described in published literature, software must be made available to editors and reviewers. We strongly encourage code deposition in a community repository (e.g. GitHub). See the Nature Portfolio guidelines for submitting code & software for further information.

## Data

Policy information about availability of data

All manuscripts must include a data availability statement. This statement should provide the following information, where applicable:

- Accession codes, unique identifiers, or web links for publicly available datasets
- A description of any restrictions on data availability
- For clinical datasets or third party data, please ensure that the statement adheres to our policy

Microbiome sequencing data is available at GEO accession number GSE235769. All codes used in the study are available on GitHub https://github.com/yj7599/mipairgit. This study used the SILVA database (https://www.arb-silva.de/)

## Research involving human participants, their data, or biological material

Policy information about studies with human participants or human data. See also policy information about sex, gender (identity/presentation), and sexual orientation and race, ethnicity and racism.

| | |
|---|---|
| Reporting on sex and gender | N/A |
| Reporting on race, ethnicity, or other socially relevant groupings | N/A |
| Population characteristics | N/A |
| Recruitment | N/A |
| Ethics oversight | N/A |

Note that full information on the approval of the study protocol must also be provided in the manuscript.

# Field-specific reporting

Please select the one below that is the best fit for your research. If you are not sure, read the appropriate sections before making your selection.

- [x] Life sciences
- [ ] Behavioural & social sciences
- [ ] Ecological, evolutionary & environmental sciences

For a reference copy of the document with all sections, see nature.com/documents/nr-reporting-summary-flat.pdf

# Life sciences study design

All studies must disclose on these points even when the disclosure is negative.

| | |
|---|---|
| Sample size | Subjects were 12 male and 11 female rhesus monkeys aged 7 to 14 years (M = 13.93 ± 1.14). Groups were matched based on sex-, age-, body weight-, and fasting blood glucose assessed at baseline. The sample size was determined based on accessibility of sex and age matched animals. |
| Data exclusions | No animals or data were excluded. |
| Replication | The overall experiment was not reproduced due to the challenges of coordinating a dietary intervention on higher order primates of this sample size. When possible, measurements were performed in replicates to mitigate technical variation. |
| Randomization | Animals were allocated based on sex-, age-, body weight-, and fasting blood glucose assessed at baseline. |
| Blinding | Metabolomics and 16S RNA sequencing were performed blinded. Data analysis and processing was performed with the same algorithms for all samples. |

# Reporting for specific materials, systems and methods

We require information from authors about some types of materials, experimental systems and methods used in many studies. Here, indicate whether each material, system or method listed is relevant to your study. If you are not sure if a list item applies to your research, read the appropriate section before selecting a response.

## Materials & experimental systems

| n/a | Involved in the study | |
|---|---|---|
| [ ] | [x] | Antibodies |
| [x] | [ ] | Eukaryotic cell lines |
| [x] | [ ] | Palaeontology and archaeology |
| [ ] | [x] | Animals and other organisms |
| [x] | [ ] | Clinical data |
| [x] | [ ] | Dual use research of concern |
| [x] | [ ] | Plants |

## Methods

| n/a | Involved in the study | |
|---|---|---|
| [x] | [ ] | ChIP-seq |
| [ ] | [x] | Flow cytometry |
| [x] | [ ] | MRI-based neuroimaging |

# Antibodies

| | |
|---|---|
| Antibodies used | CD20-APC.Cy7 (Biolegend, clone 2H7), CD3-PE.Cy7 (BD, clone SP34-2), HLA-DR-ECD (Beckman Coulter, clone Immu-357), CD14-BV421 (BD, clone M5E2), CD8-APC (Biolegend, clone SK1), CD4-FITC (BD, clone L200), CD16-PE.Cy5.5 (Biolegend, clone 3G8), CD1c-PE (Miltenyi Biotech, clone AD5-8E7), CD123-PerCP.Cy5.5 (BD, clone 9F5) and Aqua-live/dead-BV510 (ThermoFisher Scientific, L34957) |
| Validation | All Abs are either validated by the manufacturer, published manuscripts, and were all independently validated by our group.<br>For individual validation data please see the following:<br>-CD20-APC.Cy7, Biolegend clone 2H7 - validated for flow cytometry against Rhesus (https://www.biolegend.com/de-at/products/apc-anti-human-cd20-antibody-557?GroupID=BLG7906)<br>-CD3-PE.Cy7, BD clone sp34-2 - validated for flow cytometry against Rhesus (https://www.bdbiosciences.com/en-us/products/reagents/flow-cytometry-reagents/research-reagents/single-color-antibodies-ruo/pe-cy-7-mouse-anti-human-cd3.557749)<br>-HLA-DR-ECD, BD clone immu-357 - validated for flow cytometry against Rhesus (https://www.beckman.com/reagents/coulter-flow-cytometry/antibodies-and-kits/single-color-antibodies/hla-dr/im3636)<br>-CD14-BV421, BD clone M5E2 - validated for flow cytometry against Rhesus (https://www.bdbiosciences.com/en-us/products/reagents/flow-cytometry-reagents/research-reagents/single-color-antibodies-ruo/bv421-mouse-anti-human-cd14.565283)<br>-CD8-APC, Biolegend clone SK1 - validated for flow cytometry against Rhesus (https://www.biolegend.com/en-gb/cell-health/pe-anti-human-cd8-antibody-6247)<br>-CD4-FITC, BD clone L200 - validated for flow cytometry against Rhesus (https://www.bdbiosciences.com/en-us/products/reagents/flow-cytometry-reagents/research-reagents/single-color-antibodies-ruo/purified-mouse-anti-human-cd4.550625)<br>-CD16-PE.Cy5.5, Biolegend clone 3G8 - validated for flow cytometry against Rhesus (https://www.biolegend.com/nl-nl/products/pe-cyanine5-anti-human-cd16-antibody-570?GroupID=BLG8465)<br>-CD1c-PE, Militenyi Biotech clone AD5-8E7 - validated for flow cytometry against Human (https://www.miltenyibiotec.com/US-en/products/cd1c-bdca-1-antibody-anti-human-ad5-8e7.html#conjugate=vio-bright-fitc:size=100-tests-in-200-ul)<br>-CD123-PerCP.Cy5.5, BD clone 9F5 - validated for flow cytometry against Human (https://www.bdbiosciences.com/en-us/products/reagents/flow-cytometry-reagents/research-reagents/single-color-antibodies-ruo/pe-cy-5-mouse-anti-human-cd123.551065) |

# Animals and other research organisms

Policy information about studies involving animals; ARRIVE guidelines recommended for reporting animal research, and Sex and Gender in Research

| | |
|---|---|
| Laboratory animals | Subjects were 12 male and 11 female rhesus monkeys aged 7 to 14 years (M = 13.93 ± 1.14). All animals were housed at the NIA primate animal facility |
| Wild animals | No wild animals were used in this study |
| Reporting on sex | Both sexes were used in the study and allocated equally between groups. |
| Field-collected samples | No field collected samples were used in this study |
| Ethics oversight | The NIH/NIA animal experimental section is supervised by the IACUC, all experimental protocols were approved under protocol # 434-TGB-2024. |

Note that full information on the approval of the study protocol must also be provided in the manuscript.

# Flow Cytometry

## Plots

Confirm that:

- [x] The axis labels state the marker and fluorochrome used (e.g. CD4-FITC).
- [x] The axis scales are clearly visible. Include numbers along axes only for bottom left plot of group (a 'group' is an analysis of identical markers).
- [x] All plots are contour plots with outliers or pseudocolor plots.
- [x] A numerical value for number of cells or percentage (with statistics) is provided.

## Methodology

| | |
|---|---|
| Sample preparation | Blood samples were obtained under ketamine (7 – 10mg/kg, IM) or telazol (3 – 6mg/kg, IM) following an overnight fast. A 4mL sample of whole blood was kept in an EDTA until processing by ACK treatment to remove RBCs. Samples were washed twice and stained with the indicated Ab cocktail (all Abs at 1:100 concentration) for 30 min on ice. Samples were then washed and resuspended in PBS for flow cytometry |
| Instrument | BD FACSAria Fusion |
| Software | All data was collected using Diva software and analyzed using FlowJo (10.0) |

| | |
|---|---|
| Cell population abundance | No cells were sorted - all presented results are as % of population out of all live cells as indicated in the suppl. Fig. 5 |
| Gating strategy | Gating strategy is detailed in Suppl. Fig. 5 |

☒ Tick this box to confirm that a figure exemplifying the gating strategy is provided in the Supplementary Information.
